# Supplementary material for: Adaptive responses of histone modifications to resistance exercise in human skeletal muscle
Source: PLoS One. 2020 Apr 9;15(4):e0231321. doi: 10.1371/journal.pone.0231321 (PMC7145008; doi:10.1371/journal.pone.0231321)

Original images of western blotting analysis:

Left 3 blots show pre-group (subject #1-3), right 3 blots show post-group (subject #1-3).

H3.3 (Experiment 1)

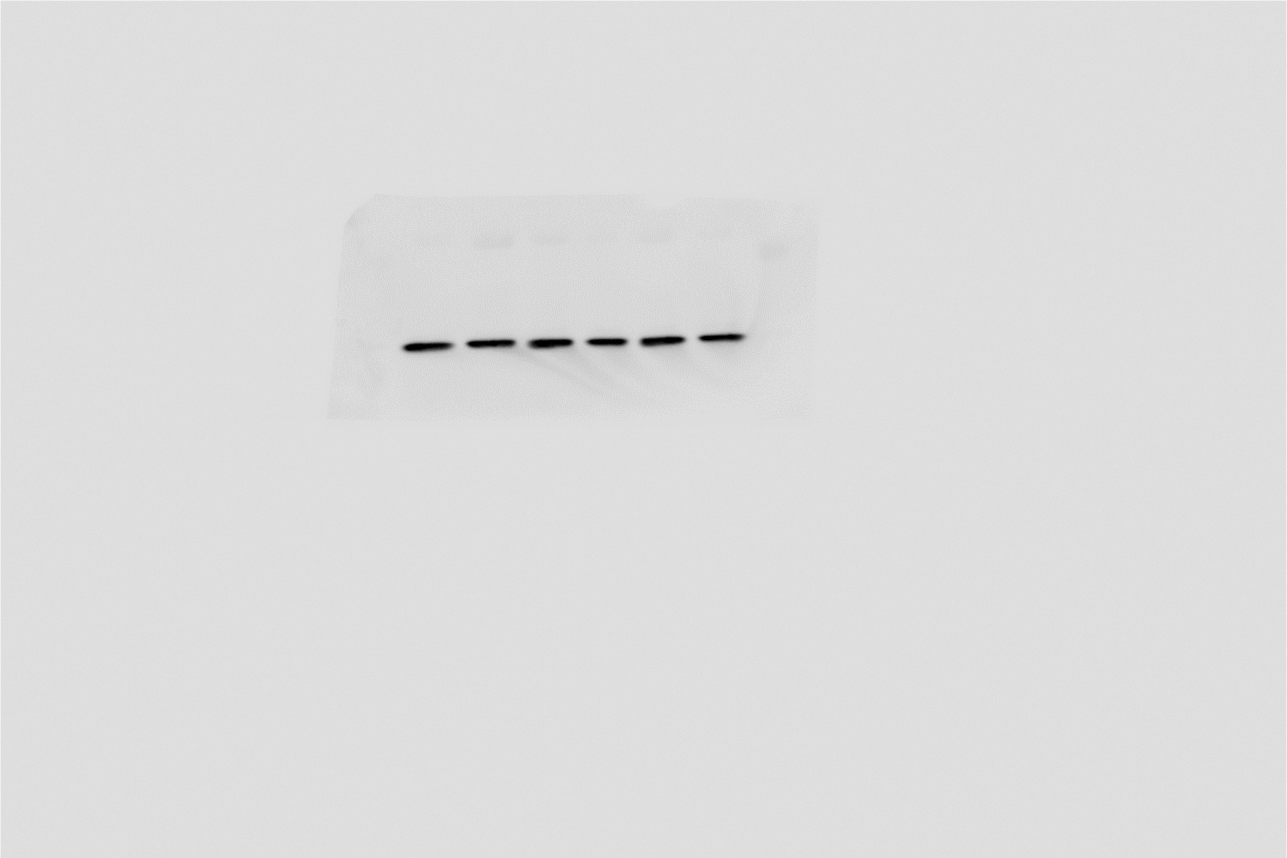

Pan-acetyl H3 (Experiment 1)

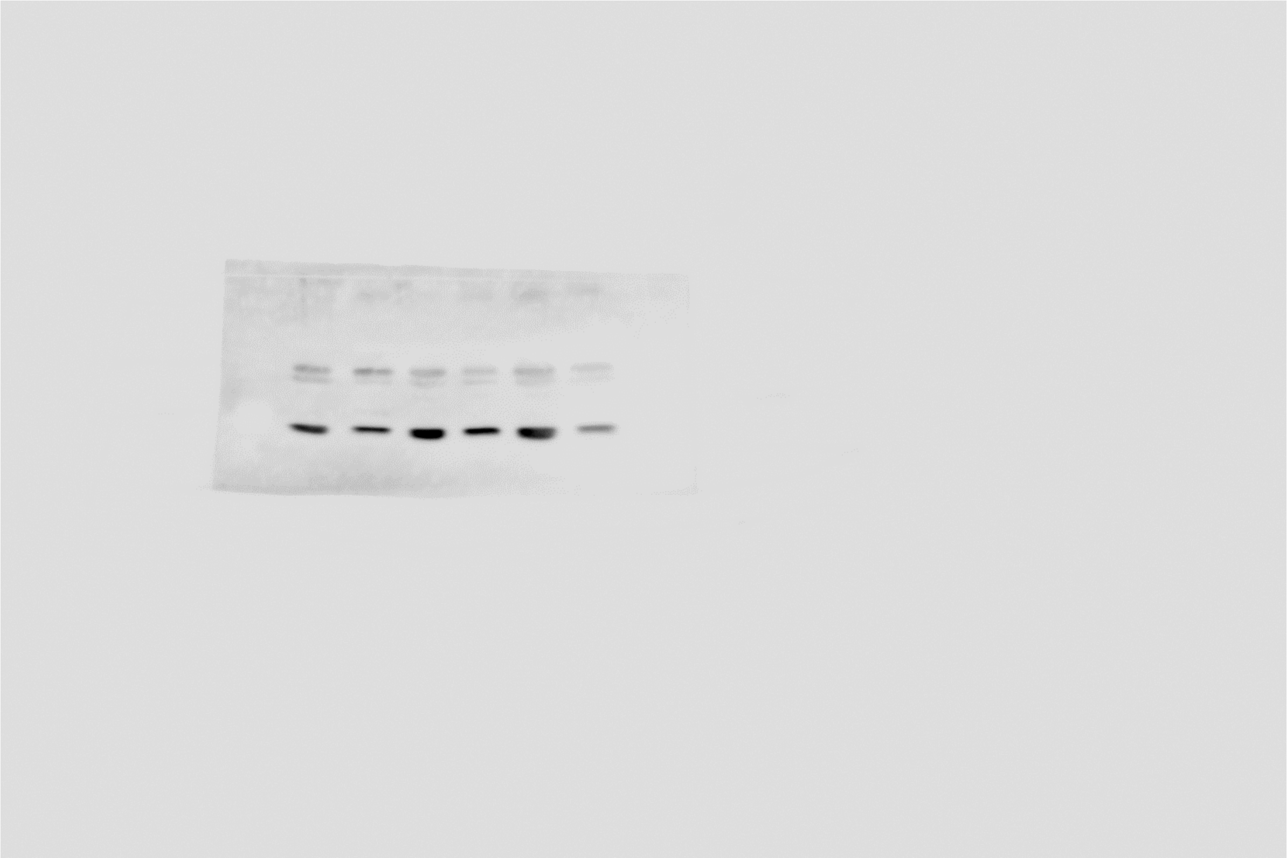

H3K4me1 (Experiment 1)

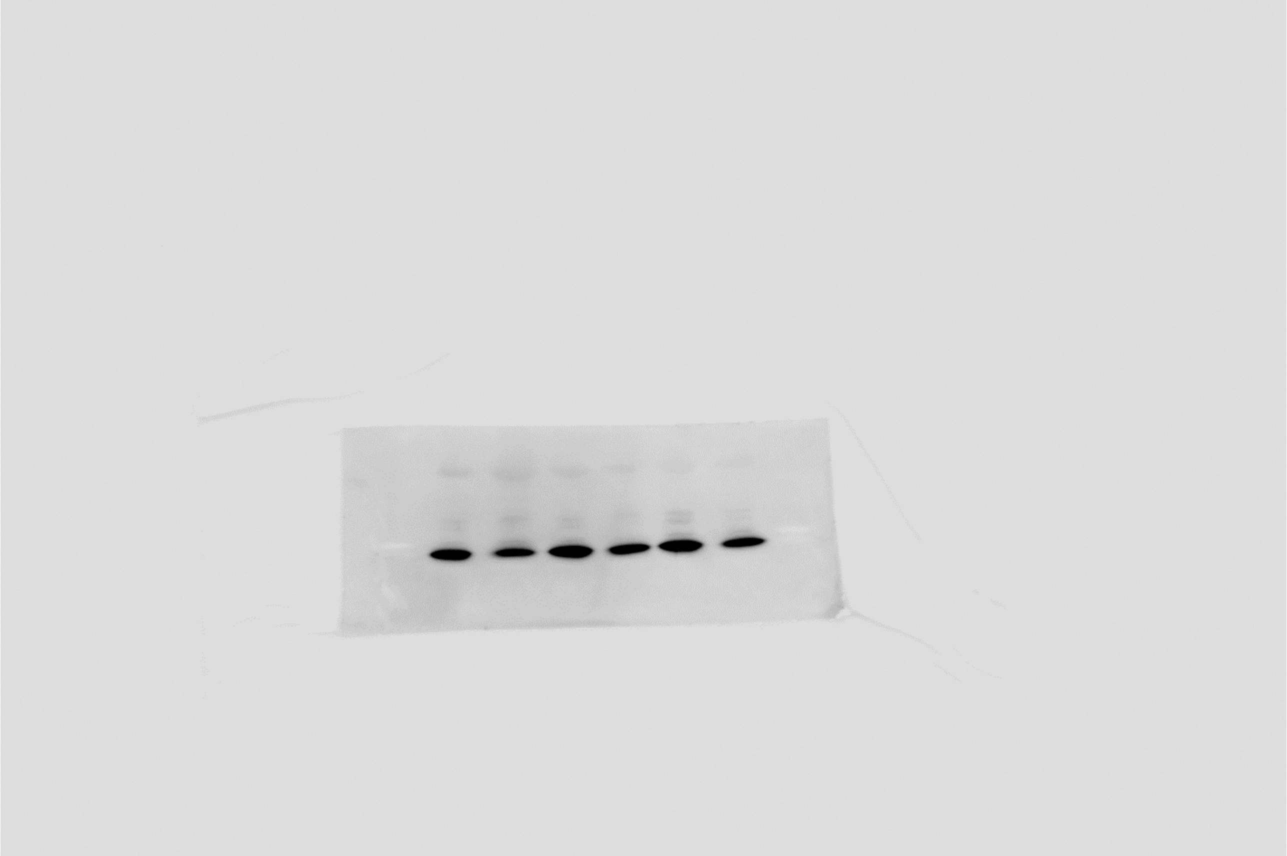

H3K27me3 (Experiment 1)

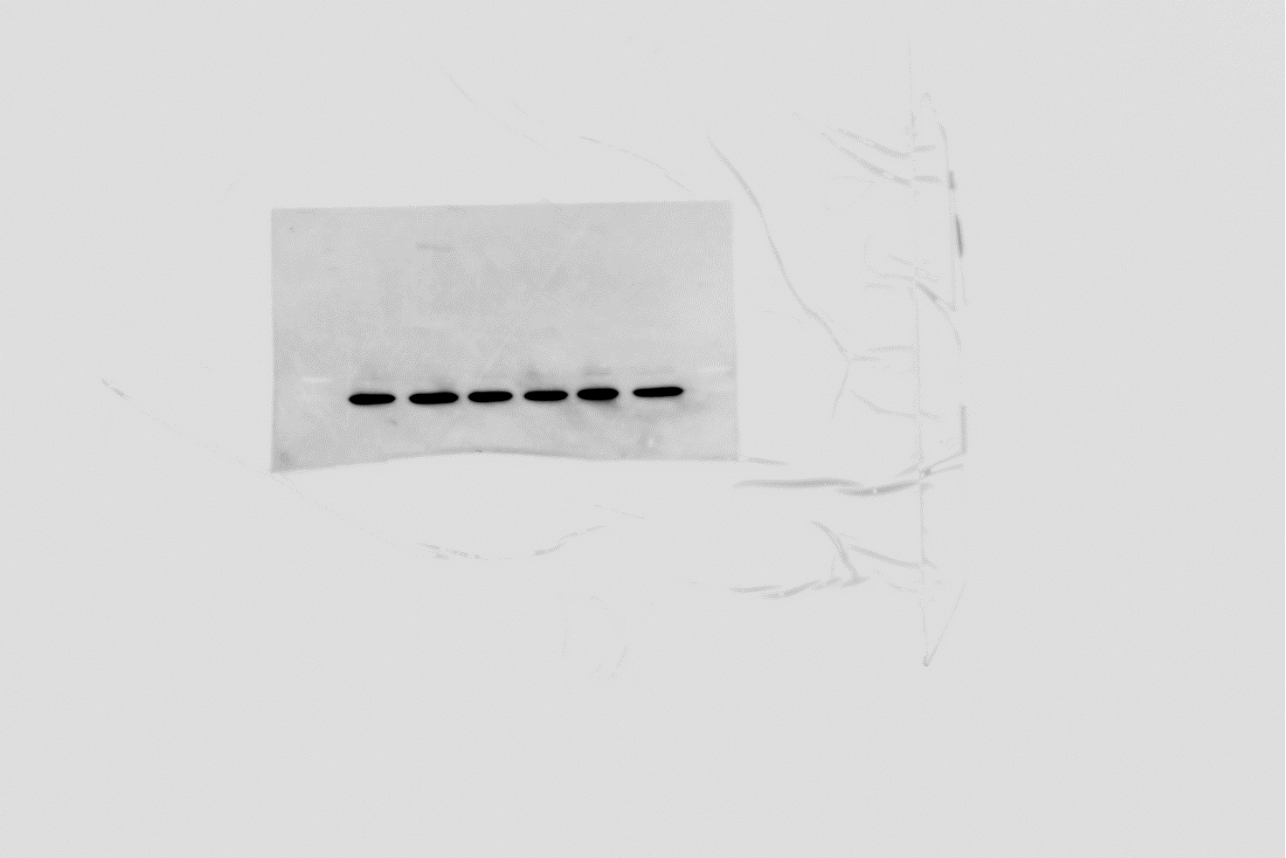

Total H3 (Experiment 1)

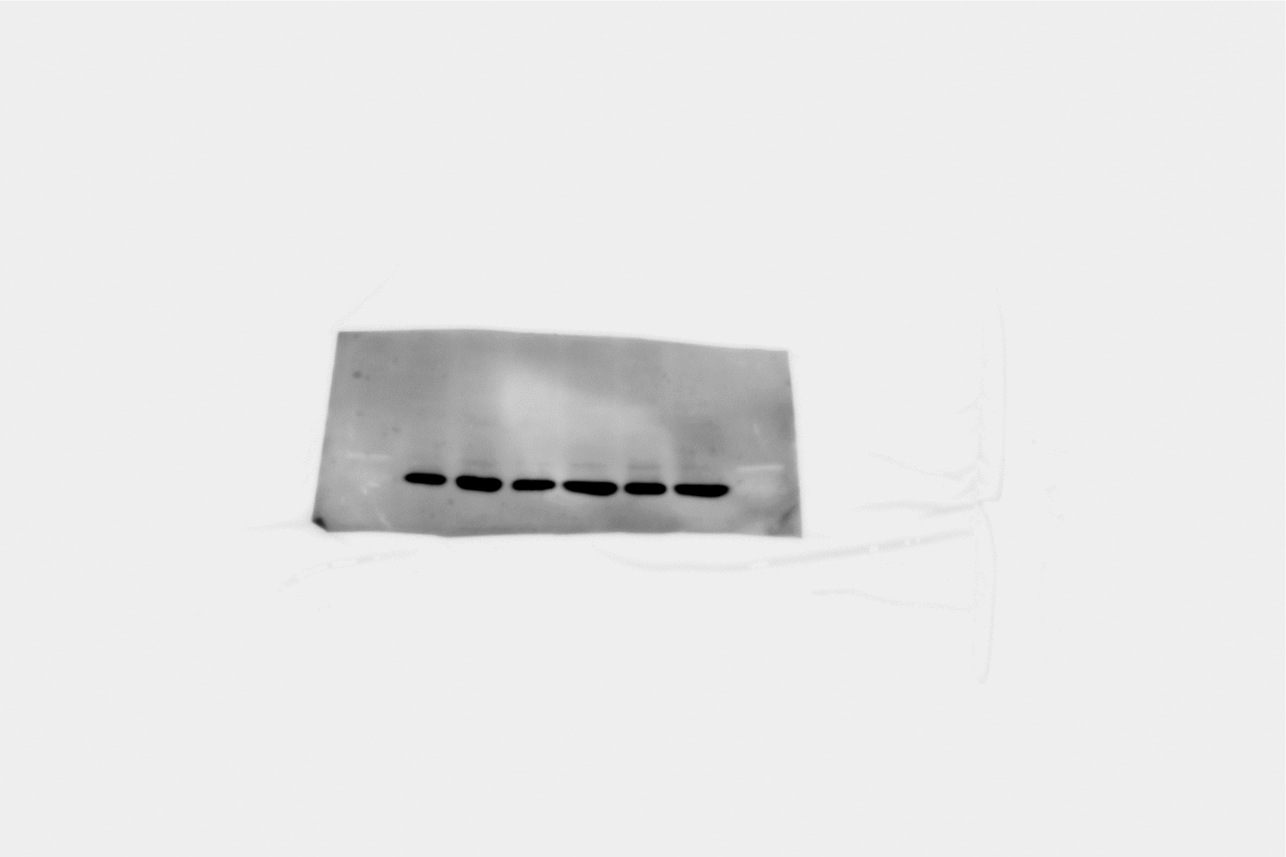

H3.3 (Experiment 2)

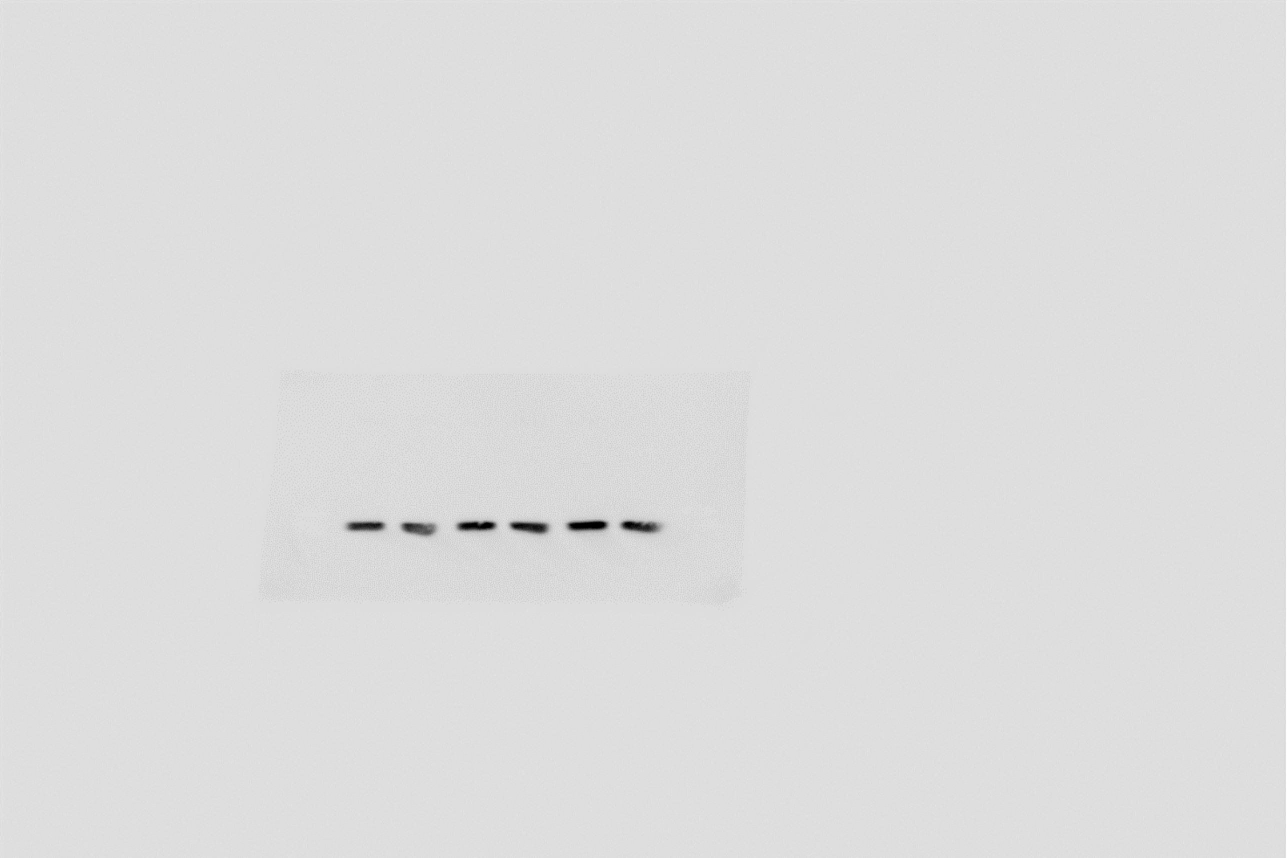

Pan-acetyl H3 (Experiment 2)

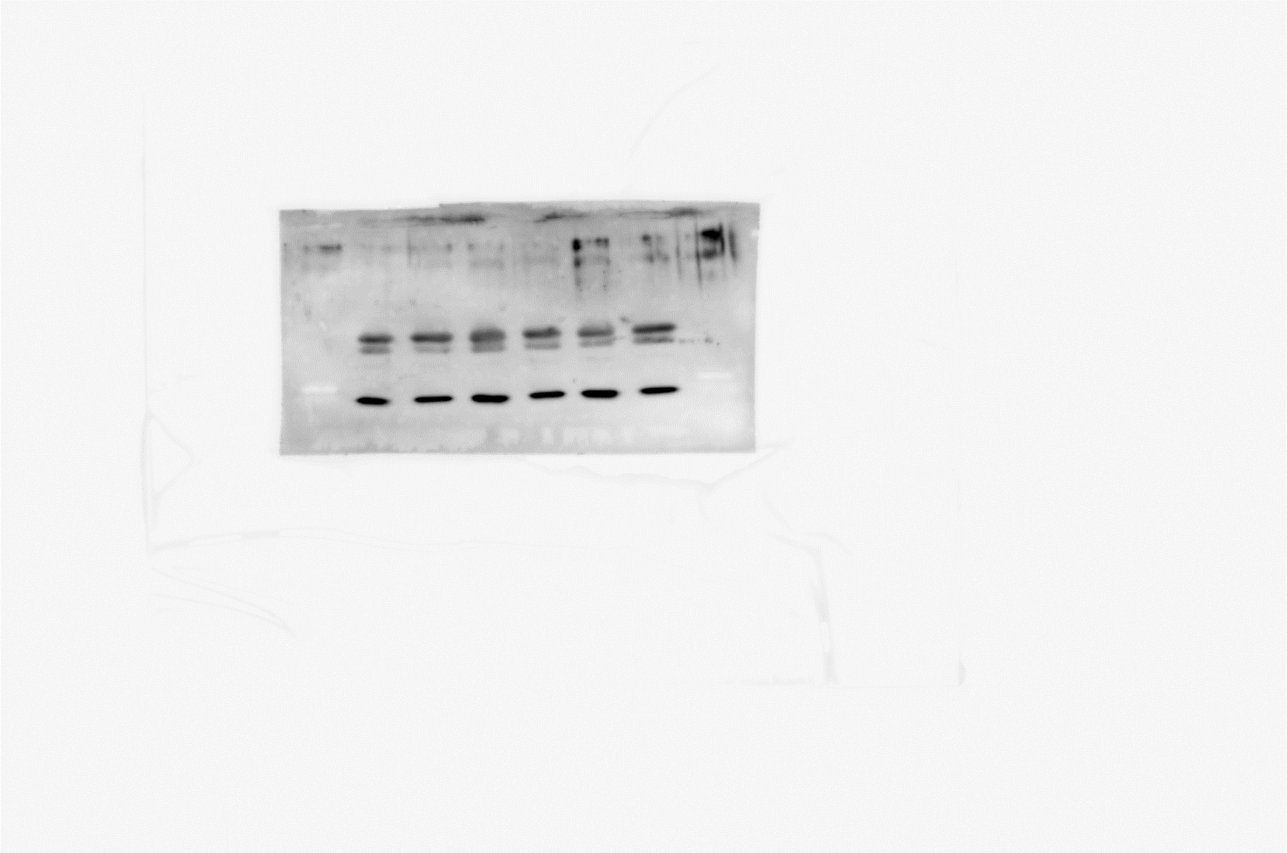

H3K4me1 (Experiment 2)

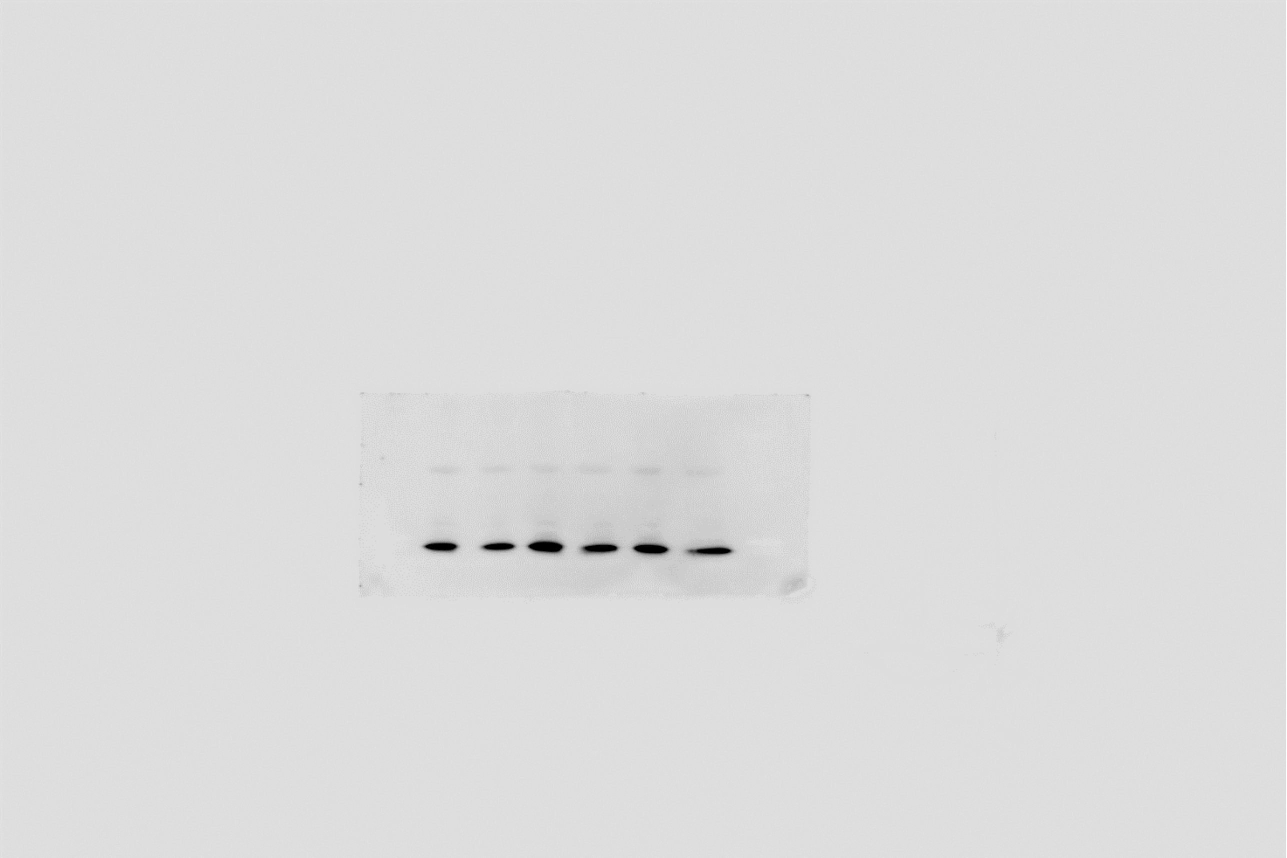

H3K27me3 (Experiment 2)

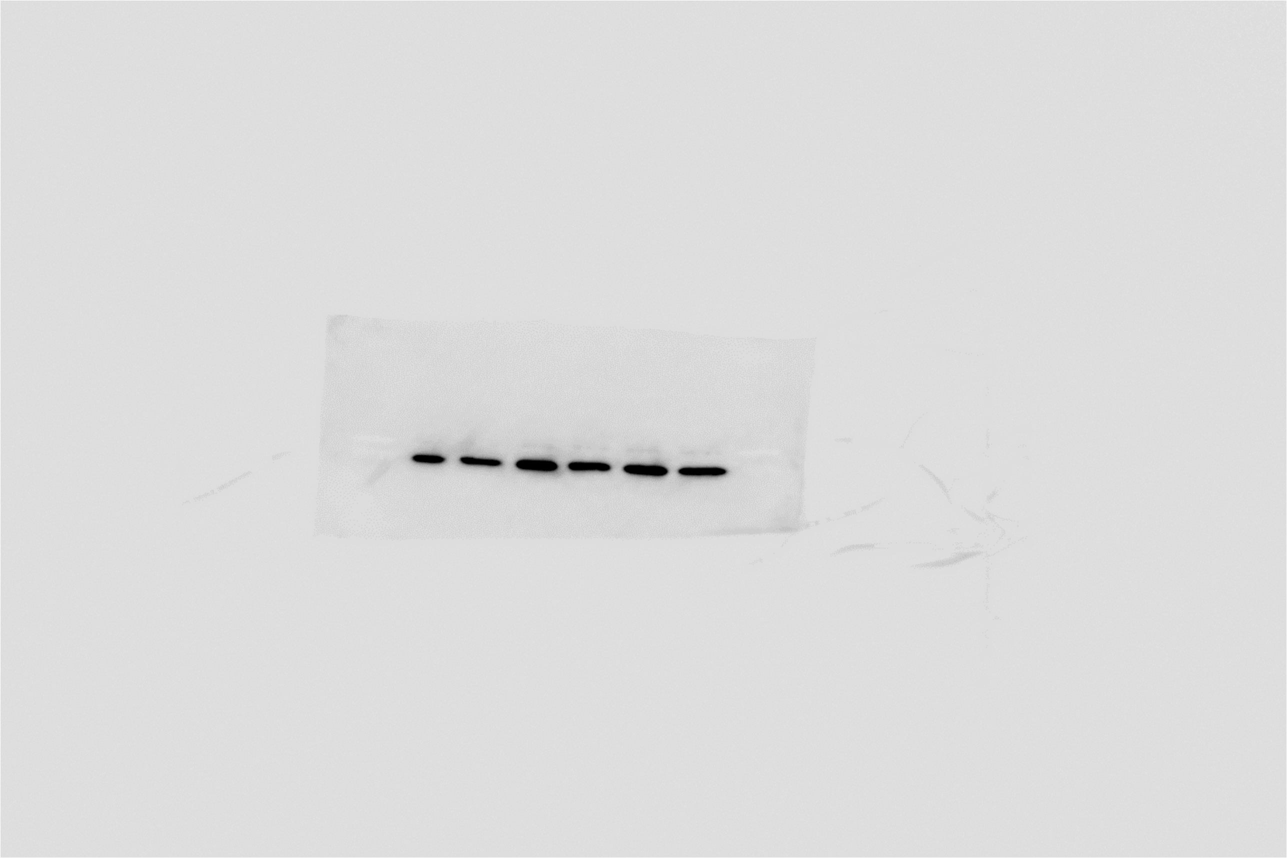

Total H3 (Experiment 2)

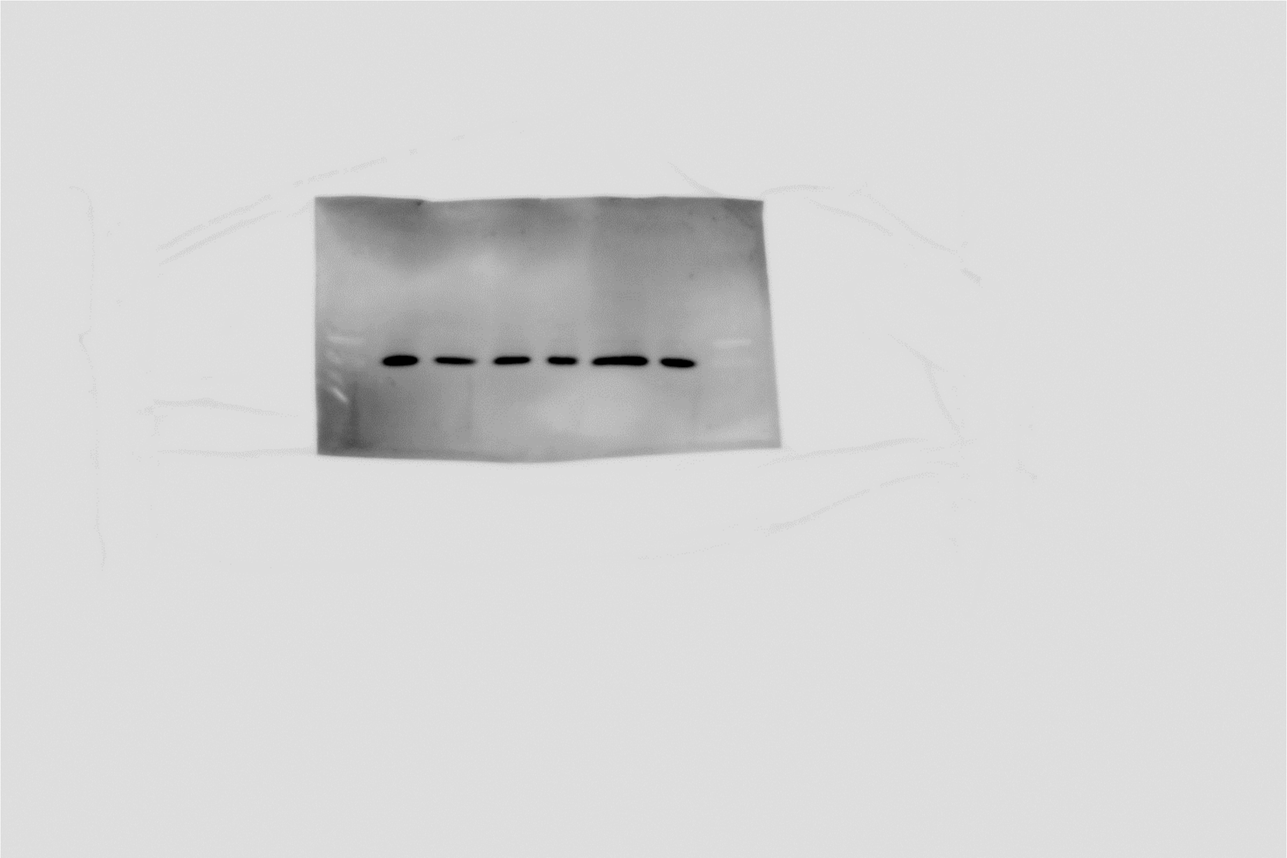

Supplement: S1 Raw images — (PDF) [file pone.0231321.s004.pdf]
