# Supplementary material for: Adaptive responses of histone modifications to resistance exercise in human skeletal muscle
Source: PLoS One. 2020 Apr 9;15(4):e0231321. doi: 10.1371/journal.pone.0231321 (PMC7145008; doi:10.1371/journal.pone.0231321)
Supplement: Attachment — Submitted filename: Responses to Reviewer 2 12-23-2019.docx [file pone.0231321.s005.docx]

Responses to the Reviewer #2

Responses to the general comments:

Thank you for positive comments. As pointed by the reviewer, manuscript was revised as that acute RE and RE training were completely separated into two different experiments. Sentence in introduction was revised as “Therefore, the present study built upon the animal literature, and aimed to investigate the response of gene expression profiles and histone modifications in human muscle before and after 1) acute RE and 2) chronic RE training.” in line 2-5 of page 4. Experimental designs were shown in methods as “

*Experiment 1*

*Subjects:* Nine male weight lifters (age=20.5±4.3yr, BMI=28.0±6.8kg/m^2^, mean ± SD) participated in Experiment 1 (acute RE). All subjects were national caliber weightlifters of H City team including a London Olympic medalist. They had been training at least 7 years and had a similar living condition such as diet, nutrition and living in the same dormitory.

*Exercise protocol:* A week prior to the test, subjects completed a 10 repeated maximum (RM) squat and bench press test to ensure appropriate exercise intensity (172.2±38kg, 82.6±16.4kg, 1RM of squat and bench press, mean ± SD, respectively). Intensity of exercise was then set at 60% of their 1RM weight and subjects competed 3 sets of 6 repetitions during each session of exercise. RE consisted of squat, single leg lunge, and deadlift which was repeated twice by all subjects.

*Collection of muscle biopsy samples:* Muscle biopsy samples were obtained using local anesthesia (1% lidocaine) administrated into the mid belly of the vastus lateralis muscle immediately before (pre-acute RE), and 3 hours (post-acute RE) after exercise. Muscle biopsy samples were frozen in liquid nitrogen and stored at −80°C until analysis. Nine biopsy samples were combined to analyze gene expressions and histone modifications in Experiment 1. Further, 3 of 9 biopsies were selected to analyze histone modifications. Throughout the analysis of biopsies obtained from Experiment 1, biopsies of pre- and post-acute RE were selected from same subjects.

*Experiment 2*

*Subjects:* Effects of RE training were examined in 21 males (age=23.7±2.5yr, BMI=24.2±2.7kg/m^2^) who had no special medical disorders in musculoskeletal, cardiovascular, and respiratory systems and had not done any regular resistance exercise in the last 2 years. The subjects were separated into 3 groups; 80FAIL (n=7, age=24.5±1.8yr, BMI=25.9±3.9kg/m^2^), 30WM (n=7, age=23.1±2.0yr, BMI=25.0±3.1kg/m^2^), and 30FAIL (n=7, age=23.0±1.2yr, BMI=24.4±1.3kg/m^2^).

*Exercise protocol:* All subjects performed 3 repeated sets of leg press, leg extension, and leg curl three times per week for 10 weeks in Experiment 2. Considering the risk of injury to subjects who had not performed resistance exercise before, 1RM was determined by using the indirect measurement method suggested in a previous report [14]. The 80FAIL group exercised at 80% of 1RM until they could not keep producing muscular contractions in every set, the 30WM group performed the total workload matching that of the 80FAIL, and the 30FAIL group exercised at 30% of 1RM until failure. For example, total work volume performed in leg press was 2,358-3,030, 2,495-3,060, and 2,995-3,588 kg/set in 80FAIL, 30WM, and 30FAIL, respectively (see Ref. 12 for more details).

*Collection of muscle biopsy samples:* Muscle biopsy was sampled from the mid belly of the vastus lateralis muscle by aforementioned procedures prior to training (pre-RE training), and 72 hours after the final session of RE (post-RE training). Nine biopsy samples were randomly collected from 3 groups (21 subjects) as samples from individuals subjected to RE training, and were used to analyze gene expressions in the present study, because the main effect test by two-way ANOVA tested in all groups found difference in muscle fiber size by time (pre vs. post) (see Ref. 12 for precise results of each group). Further, 3 of 9 biopsies were selected to analyze histone modifications. Throughout the analysis of biopsies obtained from Experiment 2, biopsies of pre- and post-RE training were selected from same subjects.” in line 5 of page 5 to line 19 of page 6.

Comments:

Please add page numbers to make review more efficient.

Responses:

We are sorry to miss page numbers. The revised version of manuscript was added page numbers.

Comments:

Please indicate the number of subjects, age, height, and body mass of each group of men. Example: “Healthy male adults were assigned to acute RE (n= , age= y, BIM= kg/m2) or RE training (n= , age= y, BIM= kg/m2) groups.”

Responses:

Physical information of subjects were added in abstract as “Healthy male adults were assigned to acute RE (n=9, age=20.5±4.3yr, BMI=28.0±6.8kg/m^2^) or RE training (n=21, age=23.7±2.5yr, BMI=24.2±2.7kg/m^2^) groups.” in line 5-7 of page 2.

Comments:

Please add more informative data to the results sections of the abstract. Please indicate the fold-increase/decrease (or percent change) of significant variables to give the readers more information up front. Example: “Up-regulation of acetylated histone 3 (H3) (+235%) and H3 …”.

Responses:

Abstract was revised to include more informative data as “RNA sequencing analysis revealed that 153 genes with GO terms including muscle development, stress response, metabolism, cell death, and transcription factor were significantly up-regulated (+291% vs. pre-acute RE) upon acute RE. Expressions of these genes were also greater (+9.6% vs. pre-RE training, p<0.05) after RE trained subjects. Significant up-regulation of acetylated histone 3 (H3) (+235%) and H3 mono-methylated at lysine 4 (+290%) and tri-methylated at lysine 27 (+849%), and down-regulation of H3.3 variant (−39%) distributions relative to total H3 were observed at transcriptionally activated loci after acute RE compared to pre-acute RE levels. Interestingly, the distribution of acetylated H3 was found to be up-regulated as compared to the level of total H3 after RE training (+40%, p<0.05).” in line 8-17 of page 2.

Comments:

Paragraph 1, Line 3: Amend sentence to: “…muscle fiber hypertrophy induced upon by resistance exercise (RE) training.”

Responses:

The sentence was revised to “Bammam et al. [1] reported that subjects were classified into extreme responders, modest responders, and non-responders depending on the magnitude of muscle fiber hypertrophy induced upon by resistance exercise (RE) training.” in line 2-5 of page 3.

Comments:

Paragraph 2: It is described here that different fiber types show different histone modification patterns. Fiber type distributions differ significantly among humans (even in the same muscle), and fiber type was not reported in the current article. Homogenizing samples with different fiber type proportions could have confounding effects on the data. In future studies, fiber type should be reported between pre vs post RE training groups, or single fibers should be isolated and pooled to get a more accurate representation of what is happening at the muscle cell level (see this paper that did fiber type specific analysis of DNA methylation in human muscle: (Begue et al. 2017). The limitation of analyzing homogenized samples should be mentioned in the Methods or discussion.

Responses:

Sentences were added in second paragraph of introduction as “Begue et al. [6] demonstrated fiber type-specific DNA methylation in human skeletal muscle, showing CpG sites of genes selectively expressed in type 1 or IIa myosin heavy chain fibers were hypomethylated. These data suggested that epigenetic regulation based on muscle fiber type characteristics affected the responsiveness of genes to exercise.” in line 20-23 of page 3.

New sub-section “Limitation of analysis” was also added in discussion, and stated as “The present study shared human biopsy samples with the original research group, whose data were published in Ref. 12 and 13. Since scientific significance would be maximized if histone modifications were measured using the samples that was enough analyzed in histochemical characteristics, and protein expressions, the present study was motivated to analyze the remaining biopsy samples. However, because of limited volume of samples remaining, we could not analyze all samples individually. Therefore, 9 biopsies were randomly chosen in Experiment 2, furthermore, 3 of 9 biopsies were selected for ChIP and western blot analysis in both experiments. But, the biopsies of pre- and post-groups in both Experiment 1 and 2 were selected from same subjects.

Epigenetic regulation differs between muscle fiber types [6]. For an essential manner analyzing data, in the present study histone modifications should have analyzed in single muscle fibers. However, that was technically difficult because the analysis needed much tissue volume. Therefore, the results of the present study discuss as changes of whole muscle homogenate including both slow- and fast-twitch fibers.” in line 2-14 of page 12.

Comments:

In the last paragraph of the introduction, there is a disconnect between going from animal studies, to human studies. I feel that a sentence or two need to be added stressing that very few studies have been conducted investigating epigenetics in human muscle after acute RE and/or RE training.

Responses:

Thank you for suggesting the example of sentences. The last paragraph of introduction was revised as “We further reported that endurance exercise training decreased the responsiveness of genes to muscular unloading in association with enhanced incorporation of histone variant H3.3 into nucleosomes in the plantaris muscle of rats. This indicated that endurance exercise training stimulated turnover of histones [7], but it is currently unclear how RE affects the distribution of histones and their modification patterns in animal models. Furthermore, even less research has been conducted on the epigenetic response to RE in human skeletal muscle [8-11]. Therefore, the present study built upon the animal literature, and aimed to investigate the response of gene expression profiles and histone modifications in human muscle before and after 1) acute RE and 2) chronic RE training.” in line 24 of page 3 to line 5 of page 4.

Comments:

Please indicate the static at software package used to analyze these data.

Responses:

Statistics section was revised as “Statistical analysis was performed using BellCurve for Excel (Social Survey Research Information Co., Ltd.). For the data analysis of RNA-seq, all FPKM values were compared in same genes between all experimental groups, and calculate a correlation coefficient (R^2^) (Fig. 1). FPKM values were normalized using the median within each gene, and averaged in up- or down-regulated genes (Fig. 2). For ChIP data, a boxplot was used to display the distribution of the data obtained from each gene (Figs. 5 and 6). Values plotted in the figures 2, 5, 6, and 7 were compared to determine the significant differences only between pre- and post-acute RE or RE training. Because the data of pre- and post-groups were obtained from same subjects in both Experiment 1 and 2, significant differences were examined using a paired *t*-test. Differences were considered significant at p<0.05.” in line 21 of page 9 to line 3 of page 10.

Comments:

Paragraph 1: Add a period after this sentence: “however, are reportedly up- or downregulated

in hypertrophic or atrophic conditions in skeletal muscles of rodents [14-17].”

Responses:

The sentence was revised as “For example, stress-responsive proteins coded by genes such as *Cryab*, *Dnajb*, *Hspa1a* and *Hspb1* function as molecular chaperones, however, are reportedly up- or down-regulated in hypertrophic or atrophic conditions in skeletal muscles of rodents [19-22].” in the bottom line of page 12.

Comments:

Write “RE Training” throughout all figures (to be similar to “Acute AE”).

Responses:

“Training” was reworded to “RE training” throughout all figures.
