# Supplementary material for: Adaptive responses of histone modifications to resistance exercise in human skeletal muscle
Source: PLoS One. 2020 Apr 9;15(4):e0231321. doi: 10.1371/journal.pone.0231321 (PMC7145008; doi:10.1371/journal.pone.0231321)
Supplement: Attachment — Submitted filename: Responses to Reviewer 2 2-22-2020.docx [file pone.0231321.s006.docx]

Responses to Reviewer 2:

Thank you for your advice for English grammar of our manuscript. Grammatical errors were corrected using a commercial English editing service. Certificate of the service is also attached to this revision.

Comment:

Remove "in the": "Exercise training causes epigenetic changes in skeletal muscle, although it is unclear how resistance exercise (RE) affects in the histone modifications."

Response:

The sentence was revised to “Exercise training causes epigenetic changes in skeletal muscle, although it is unclear how resistance exercise (RE) affects histone modifications.” in line 2-3 of page 2.

Comment:

Should be "alters": "These results indicated that a single bout of RE drastically alter both

gene expressions and histone modifications in human skeletal muscle."

Response:

The sentence was revised to “These results indicate that a single bout of RE drastically alters both gene expressions and histone modifications in human skeletal muscle.” in line 17-18 of page 2.

Comment:

Add "is" to this sentence: " It was also suggested that enhanced histone acetylation closely related to up-regulation of gene expressions after RE training."

Response:

The sentence was revised to “It is also suggested that enhanced histone acetylation is closely related to up-regulation of gene expressions after RE training.” in line 18-19 of page 2.

Comment:

A lot of the "Limitations of analysis" section needs to be reworded to be grammatically correct. Example sentence that does not make sense: "Since scientific significance would be maximized if histone modifications were measured using the samples that was enough analyzed in histochemical characteristics, and protein expressions, the present study was motivated to analyze the remaining biopsy samples."

Response:

This sentence was deleted from the Discussion.

Comment:

The section titled "Fundings" should just be "Funding".

Response:

The section title was corrected to “FUNDING” in page 17.
